# Comparison of Ferguson and Gross herniotomy with Mitchell Banks’ herniotomy in boys older than two years

**DOI:** 10.12669/pjms.37.1.3216

**Published:** 2021

**Authors:** Hafiz Mahmood Ahmad, Fatima Naumeri, Usama Saud, Ghazala Butt

**Affiliations:** 1 Dr. Hafiz Mahmood Ahmad, MBBS. Department of Pediatric Surgery, King Edward Medical University/Mayo Hospital Lahore, Pakistan; 2 Dr. Fatima Naumeri, MCPS, FCPS. Department of Pediatric Surgery, King Edward Medical University/Mayo Hospital Lahore, Pakistan; 3 Dr. Usama Saud, MBBS. Department of Pediatric Surgery, King Edward Medical University/Mayo Hospital Lahore, Pakistan; 4 Dr. Ghazala Butt, MD, PhD. Department of Dermatology, King Edward Medical University/Mayo Hospital Lahore, Pakistan

**Keywords:** Inguinal Hernia, Children, Boys, Mitchell-Banks’, Herniotomy, Ferguson and Gross Herniotomy, Recurrence

## Abstract

**Background & Objective:**

In children younger than two years, most surgeons perform the inguinal herniotomy superficially through the external ring, a technique known as Mitchell-Banks’ Herniotomy (MBH) while in older children, commonly Ferguson and Gross Herniotomy (FGH) is performed which involves opening of inguinal canal. Our aim was to compare the FGH and MBH in terms of recurrence in boys with inguinal hernia.

**Methods:**

Boys with inguinal hernia presenting to Pediatric Surgery, Mayo Hospital Lahore from Dec 2016 to January 2018 were included in the study, if older than two years and younger than 14 years and without palpable deep ring (2 cm or more in width) or strangulation of inguinal hernia or malnutrition. They were randomly allocated in 2 groups after obtaining informed consent from parents, and underwent MBH (Group-A) and FGH (Group-B). Children were called for follow up after 1 week and at 6 months to assess for recurrence.

**Results:**

Total 260 patients with inguinal hernia were enrolled (NCT: 03392636). The mean age of boys in Group-A was 5.2±3.0 years and in Group-B was 5.9±3.1 years. Mean operating time in Group-A (26.65±3.22 minutes) was longer than Group-B (15.92±4.22 minutes), and scrotal oedema was noted in 38 (29.2%) cases in Group-A, while 7 (5.4%) cases in Group-B. Testicular atrophy was noted in one patient of Group-B. Recurrence occurred in 1(0.8%) patient in Group-A, and in 8(6.2%) patients in Group-B (p-value 0.018).

**Conclusion:**

Mitchell-Banks’ herniotomy has lower recurrence rate than Ferguson and Gross Herniotomy in boys older than two years.

## INTRODUCTION

Inguinal herniotomy is one of the most commonly performed procedure by a pediatric surgeon. The reported incidence of inguinal hernia in mature infants varies from 1-5% to 12-25% with a family history, and a male preponderance. It is slightly higher in pre-mature infants about 10-30%. In children, it is almost always indirect inguinal hernia caused by patent processus vaginalis. Right sided hernias are twice as common as those on the left and bilateral hernias are present approximately 10% of the times.^1^-^3^

The basic principle of herniotomy for indirect inguinal hernia is the ligation of processus vaginalis as described by Marcy in 1871.^4^,^5^ This can be achieved by different methods such as open method, laparoscopy, laparoscope assisted percutaneous extra peritoneal closure or mini-incision herniotomy.^5^-^8^

Ferguson and Gross herniotomy (FGH) and Mitchell-Banks’ herniotomy (MBH) are the two most commonly performed open methods of pediatric inguinal herniotomy. FGH involves opening of inguinal canal and herniotomy at the level of internal inguinal ring or at the level of pre-peritoneal fat. In contrast, in MBH, the same is accomplished without opening inguinal canal. Traditional teaching for inguinal herniotomy is to do MBH in children under the age of two years and FGH in older children.^2^,^3^,^9^-^11^

Most common cause of a recurrent inguinal hernia in children is failure to identify or to completely ligate the indirect inguinal hernial sac at the level of internal ring or pre-peritoneal fat.^7^ Initially it was thought that MBH in older children prevents effective ligation and leads to recurrence, yet studies indicate the inguinal canal length to be in between 4-23 mm to 40-65 mm maximum in children and by applying gentle traction on superficial ring, hernial sac can be ligated at the level of pre-peritoneal fat effectively as tissues are elastic.^9^-^12^ A study conducted by Jadhav and co-authors, mentioned the recurrence rate of FGH as 6%.^13^ In another study, the recurrence of both the techniques were 0%.^9^

The objective of this study was to compare the recurrence rate between the two different procedures of inguinal herniotomy in boys older than two years. Rationale was that no randomized trial is available to compare the two techniques and if the recurrence rates are comparable, then MBH can be recommended as in this technique, there is minimum distortion of anatomy.

## METHODS

This randomized controlled trial (Trial registration number NCT:03392636 and IRB No: 212/RC/KEMU dated 26-11-2016) was carried out in the department of Pediatric Surgery, King Edward Medical University/ Mayo Hospital Lahore from December 2016 to January 2018. Sample size of 260 cases (130 in each group), was calculated with 95% confidence level, 80% power of test with expected percentage of recurrence of MBH as 0% and FGH as 6% using formula:^13^

INLINE

Non probability convenience sampling technique was used. All boys aged between 2-14 years presenting in Pediatric Surgery Department, Mayo Hospital Lahore with inguinal hernia were included. Exclusion criteria was children who were severely malnourished(Child between z score of -2 to -3 for weight for height , and between z score of -2 to -3 for height for age and hemoglobin less than 8 g/dl), children with very large hernia needing internal ring repair (a palpable deep ring that is two cm or more in width), children with sliding hernia (hernia containing the segment of bowel or bladder as component of its sac wall) or strangulated hernia (hernia with vascular compromise and showing the signs of irreducibility and intestinal obstruction). Children with connective tissue disorders or undescended testis or associated hydrocele were also excluded.

After taking informed consent from parents, children fulfilling the criteria were randomly allocated in two groups via lottery method. Children in Group-A (Case) underwent Mitchell Banks Herniotomy and those in Group-B (Control) underwent Ferguson and Gross Herniotomy.

Under aseptic measures, inguinal skin cease incision was made in both procedures. In MBH, inguinal herniotomy was done through the superficial inguinal ring, by dissecting the hernia sac from vas deferens and vessels without opening inguinal canal and then ligating sac at level of pre-peritoneal fat by applying gentle traction on superficial inguinal ring (Fig.2). In FGH, inguinal herniotomy was performed after opening inguinal canal. Patients were discharged on same day and were called for follow up at one week to rule out any complications (secondary outcome) and six months interval post operatively to look for recurrence (primary outcome). Recurrence was labelled if there was re-appearance of reducible swelling in inguino-scrotal region assessed after six months of procedure and ultrasonography of the swelling showed abdominal contents. Doppler ultrasound and assessment of pre-operative and post-operative testicular volume was also done to rule out testicular atrophy. It was assessed by medical officer of OPD, who was blind to random assortment of patient and the type of herniotomy done.

The data was collected by the researcher himself on the prescribed proforma. Data was entered and analyzed in SPSS version 23. Quantitative variables like age were presented as mean and standard deviation. Qualitative variables like recurrence were presented as frequency and percentage. Comparison of two groups’ outcome was done by applying chi-square test and p value <0.05 was taken as significant. Effect modifiers like size of defect and age of patient were stratified and post stratification comparison of two groups was done.

## RESULTS

Total patients enrolled were 260. Out of these, 158 (60.8%) patients had right sided inguinal hernia, 90 (34.6%) had left inguinal hernia, while remaining 12 (4.6%) had bilateral inguinal hernia. Mean age of patients in Group-A was 5.2±3 years while in Group-B,the mean age for children undergoing Ferguson Gross herniotomy was 5.9±3.1 years. Mean weight of children in Group-A was 17.93±6.7 kg, while the mean weight of children in Group-B was 19.1±6.98 kg. Mean operation time in Group-A was 26.65±3.22 minutes and 15.92±4.22 minutes in Group-B. Mean size of incision in Group-A was 2.5±0.11 cm and in Group-B was 2.8±0.1 cm.

Eleven (8.5%) patients had tearing of processus vaginalis in MBH and only one (0.8%) in FGH. None of patients undergoing both techniques developed wound infection post-operatively. While 38 (29.2%) developed scrotal oedema in MBH and 7 (5.4%) developed it in FGH. Eight patients (6.2%) in Group-A developed scrotal hematoma, while 4 (3.1%) developed it in Group-B.

Recurrence was noted in 1 (0.8%) cases in MBH while it was observed in 8 (6.2%) cases in FGH, with p value of 0.018. One (0.8%) patient who underwent FGH developed testicular atrophy and no patient had testicular retraction.There was no difference in recurrence in both groups even when compared post stratification for age and size of ring, as seen in Table-I, II.

**Table-I:** Post stratification comparison of recurrence in both groups with respect to age.

*Age groups*	*Recurrence*	*Group-A*	*Group-B*	*Total*	*p-value*

		Mitchell Banks Herniotomy	Ferguson & Gross Herniotomy		
	yes	1(1%)	6(6.1%)	7(3.6%)	
≤7 years	no	98(99%)	92(93.9%)	190(96.4%)	0.053
	Total	99(100%)	98(100%)	197(100%)	
	yes	0(0%)	2(6.3%)	2(3.2%)	
>7 years	no	31(100%)	30(93.8%)	61(96.8%)	0.157
	Total	31(100%)	32(100%)	63(100%)	

**Table-II:** Post stratification comparison of recurrence in both groups with respect to size of ring.

*Size of defects*	*Recurrence*	*Group-A*	*Group-B*	*Total*	*p-value*

		Mitchell Banks Herniotomy	Ferguson & Gross Herniotomy		
	Yes	0(0%)	3(4.8%)	3(2.5%)	
≤1.5 cm	No	56(100%)	59(95.2%)	115(97.5%)	0.095
	Total	56(100%)	62(100%)	118(100%)	
	Yes	1(1.4%)	5(7.4%)	6(4.2%)	
>1.5 cm	No	73(98.6%)	63(92.6%)	136(95.8%)	0.076
	Total	74(100%)	68(100%)	142(100%)	

## DISCUSSION

Although inguinal herniotomy is a commonly performed procedure, yet different surgeons prefer different techniques and different approaches.^2^-^10^ In this study, we found that there is no need to open inguinal canal in selected children, rather MBT was safer than FGH in terms of recurrence (p value of 0.018).

In this study, 60.8% patients had right inguinal hernia while 34.6% had left inguinal hernia. Right sided inguinal hernia was found to be almost twice more common than left inguinal hernia which is very much consistent with published literature.^1^-^3^,^13^

Operation time in Mitchell Banks herniotomy was 26.65±3.22 minutes while the mean operation time in Fergusson Gross herniotomy was 15.92±4.22 min. The increased operation time may be due to less familiarity with the procedure by all surgeons especially residents, needing supervision and a need to apply traction at times, to achieve high ligation of the hernia sac. This also contributed to more tearing of processus vaginalis in MBH (11 patients) and only 1 (0.8%) developing it in FGH. Yoshimura and colleagues have reported that operative times are more for residents performing herniotomy as compared to specialists.^14^

More patients in MBH developed scrotal oedema and haematoma probably because of extra dissection and extra traction required in this technique. Thirty eight patients developed scrotal oedema (29.2%) in MBH while seven (5.4%) developed it in FGH. Eight patients (6.2%) developed scrotal haematoma in MBH while four (3.1%) developed in FGH. This is more than other such comparative studies such as study conducted by Turk et al in which the incidence of scrotal oedema and hematoma was 1% and 0.6% in FGH Group-And 0.7% and 0.6% respectively in MBH group.^11^ Similarly lower frequency was reported by Nasar and colleagues, around 5% scrotal hematoma and 2.7% scrotal oedema.^15^ Although Javaid and co-authors reported slightly higher post-operative complications (17%) with scrotal oedema 14% and scrotal hematoma 2%.^16^

One patient developed testicular atrophy in Group-B. The incidence reported is 2-3% after incarceration.^3^ Overall 30% cases of testicular atrophy occur within 1^st^ year of repair, with 5.1 cases reported in 10,000 persons per year.^17^

Recurrence was less in MBH technique (0.8%). The length of inguinal canal and wider external ring are important factors for the success of MBH in children. Studies have documented the inguinal canal length in a range of 4-23mm to a maximum of 40-50mm, an average of 0.7-1.9 cm in children younger than 13 years.^9^,^11^,^12^,^18^ Another important factor contributing to the success of this procedure is flexible fascia and less oblique inguinal canal, allowing easy traction on external ring and greater visibility of internal ring.^9^,^10^

Reported recurrence rate are relatively lower in uncomplicated inguinal hernia, around 1-2.5%. The most important factors contributing include adequate surgical training, experience, and tissue handling.^2^,^3^,^5^ Recurrence rate of FGH (6.2%) in this study is similar to the incidence of 6% reported by Dinesh Jadhav and colleagues.^13^ Nasar et al reported recurrence rate of 1.1%.^15^

Recurrence was observed for only up to six months which is a brief period because recurrence has been reported even up to 6.7 years after the procedure in a recent study with a long follow-up. The reported median time was 209 days with time interval increasing with older age, although the majority of recurrences occurred within the first year following hernia repair (60%).^19^ So a prolonged follow up is required to draw a definitive conclusion when primary outcome is taken as recurrence.

### Limitation of this study

It is short period of follow-up and conducting it in a single centre. The results may be generalized as different surgeons performed both procedures.

## CONCLUSION

Mitchell-Banks’ herniotomy has lower recurrence rate than Ferguson and Gross Herniotomy in boys older than two years.

### Authors Contribution:

**HMA:** designed, data collection, statistical analysis, final approval and accountable for manuscript

**FN:** conceived, manuscript writing, final approval and accountable for manuscript

**US:** data collection, reference writing, final approval and accountable for manuscript

**GB:** reviewed the manuscript, statistical analysis, final approval and accountable for manuscript
